# Synthesis and Antidepressant Activity Profile of Some Novel Benzothiazole Derivatives

**DOI:** 10.3390/molecules22091490

**Published:** 2017-09-07

**Authors:** Ümide Demir Özkay, Ceren Kaya, Ulviye Acar Çevik, Özgür Devrim Can

**Affiliations:** 1 Department of Pharmacology, Faculty of Pharmacy, Anadolu University, 26470 Eskişehir, Turkey; cer.kaya@anadolu.edu.tr (C.K.); ozgurdt@anadolu.edu.tr (Ö.D.C.); 2 Department of Pharmaceutical Chemistry, Faculty of Pharmacy, Anadolu University, 26470 Eskişehir, Turkey; uacar@anadolu.edu.tr

**Keywords:** activity cage, antidepressant, benzothiazole, modified forced swimming test, tail suspension test

## Abstract

Within the scope of our new antidepressant drug development efforts, in this study, we synthesized eight novel benzothiazole derivatives **3a**–**3h**. The chemical structures of the synthesized compounds were elucidated by spectroscopic methods. Test compounds were administered orally at a dose of 40 mg/kg to mice 24, 5 and 1 h before performing tail suspension, modified forced swimming, and activity cage tests. The obtained results showed that compounds **3c**, **3d**, **3f**–**3h** reduced the immobility time of mice as assessed in the tail suspension test. Moreover, in the modified forced swimming tests, the same compounds significantly decreased the immobility, but increased the swimming frequencies of mice, without any alteration in the climbing frequencies. These results, similar to the results induced by the reference drug fluoxetine (20 mg/kg, po), indicated the antidepressant-like activities of the compounds **3c**, **3d**, **3f**–**3h**. Owing to the fact that test compounds did not induce any significant alteration in the total number of spontaneous locomotor activities, the antidepressant-like effects of these derivatives seemed to be specific. In order to predict ADME parameters of the synthesized compounds **3a**–**3h**, some physicochemical parameters were calculated. The ADME prediction study revealed that all synthesized compounds may possess good pharmacokinetic profiles.

## 1. Introduction

Depression is a common and serious disability-causing mental disorder with high incidence and recurrence [1,2]. It is estimated by the World Health Organization that in the future it will become the second major cause of disability after cardiovascular diseases [2]. Depression is characterized by several clinical symptoms, including sadness, loss of interest or pleasure, disturbed sleep and appetite, feelings of tiredness, low self-esteem or poor concentration [1]. Currently used antidepressant drugs alleviate the symptoms of major depression, but these treatment approaches have many limitations such as requirement of long times to produce therapeutic responses, low response rates, and various side effects [3,4], therefore, there is a strong demand to develop novel antidepressant agents with greater efficacy and fewer adverse effects.

Benzothiazole belongs to a class of bicyclic heterocyclic compounds, formed with a fused benzene and thiazole moiety containing nitrogen and sulphur in its structure [5]. The benzothiazole core has attracted continuing interest for drug development studies, because it has demonstrated a wide spectrum of pharmacological activities such as anticancer [6,7,8], antiviral [9], antibacterial [7], antituberculosis [10], antimalarial [11], antifungal [7,12], anthelmintic [13], antileishmanial [14], antiinflammatory [15], antidiabetic [16], antioxidant [17], antiasthmatic [18], and immunomodulatory properties [19]. Apart from these activities, the benzothiazole moiety has also attracted attention in central nervous system (CNS)-related drug discovery studies. Several pharmacological activities such as analgesic [20,21], anticonvulsant [22,23], anti-Alzheimer [24,25], anticholinesterase [26,27], MAO inhibitory [28], adenosine receptor antagonist [29], and neuroprotective effects [30,31] of benzothiazole derivatives have been reported. Furthermore, there are approved drugs that contain a benzothiazole core. For example, riluzole is used for the treatment of amyotrophic lateral sclerosis. Besides, another benzothiazole-based drug, pramipexole, has been approved for the treatment of Parkinson’s disease [32]. In addition to these CNS-related pharmacological effects, various benzothiazole-based compounds have been shown to possess remarkable binding affinities to serotonin transporters, 5HT1A, and also 5HT2A receptors [33,34], which are important sites of action for antidepressant activity. The antidepressant-like activities of several benzothiazole derivatives have been shown by various animal model such as forced swimming and tail suspension tests (TST) [33,35].

Based on the previous papers reporting the antidepressant-like potential of the benzothiazole scaffold, in this study, we synthesized novel compounds containing this ring system and investigated their potential antidepressant-like effects.

## 2. Results and Discussion

### 2.1. Chemistry

**Scheme 1:**
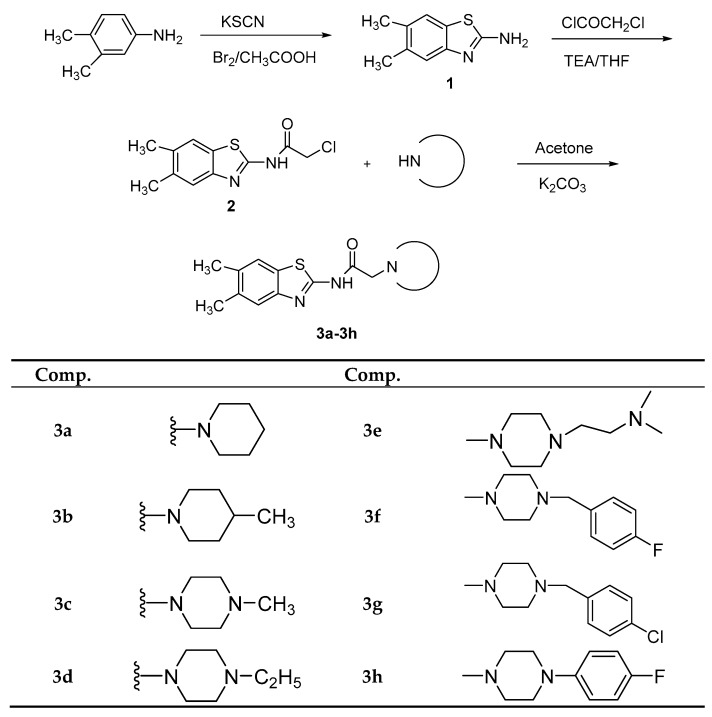
Synthesis of the compounds **3a**–**3h**.

Target molecules **3a**–**3h** were synthesized in three steps as shown in Scheme 1. Initially, 5,6-dimethylbenzo[*d*]thiazol-2-amine (**1**) were prepared via reaction of 3,4-dimethylaniline, potassium thiocyanate, and bromine. In the second step, compound **1** was acetylated with chloroacetyl chloride to afford 2-chloro-*N*-(5,6-dimethylbenzo[*d*]thiazol-2-yl) acetamide (**2**). Finally, compound **2** was reacted with piperazine or piperidine derivatives in acetone to obtain target compounds (**3a**–**3h**).

Structure elucidations of the final compounds were performed by FT-IR, ^1^H-NMR, ^13^C-NMR and HRMS spectroscopic methods. In the IR spectra, significant stretching bands due to N-H, C=O bonds at 3315–3238 cm^−1^ and 1705–1685 cm^−1^, respectively, were observed. The stretching absorption belonging to the 1,4-disubstituted benzene unit was determined at 839–831 cm^−1^. In the ^1^H-NMR spectra, N-H and -CH_2_ protons in the amide group observed as a singlet at 12.01–11.84 ppm and 3.38–3.01 ppm, respectively. The -CH_3_ protons gave a singlet peak between 2.24–2.32 ppm. The C4 and C7 protons of the benzothiazole ring gave peaks at 7.70–7.36 and 7.53–7.20 ppm as a singlet, respectively. In the ^13^C-NMR spectra carbon of C=O group was assigned to the peaks at 169.62–171.27 ppm. All measured mass and isotope scores were compatible with calculated values for the compounds **3a**–**3h**.

### 2.2. Pharmacology

In this study, based on the antidepressant activity potential of benzothiazole-based compounds, we searched for potential antidepressant-like effects of the newly synthesized benzothiazole derivatives **3a**–**3h** through TST and modified forced swimming tests (MFST). Activity cage tests were also incorporated into the experimental protocol to evaluate any possible effect of the test compounds on the spontaneous locomotor activities of mice. Test compounds were administered to the mice at a dose of 40 mg/kg, which was previously reported for benzothiazole-based compounds [33].

**Figure 1:**
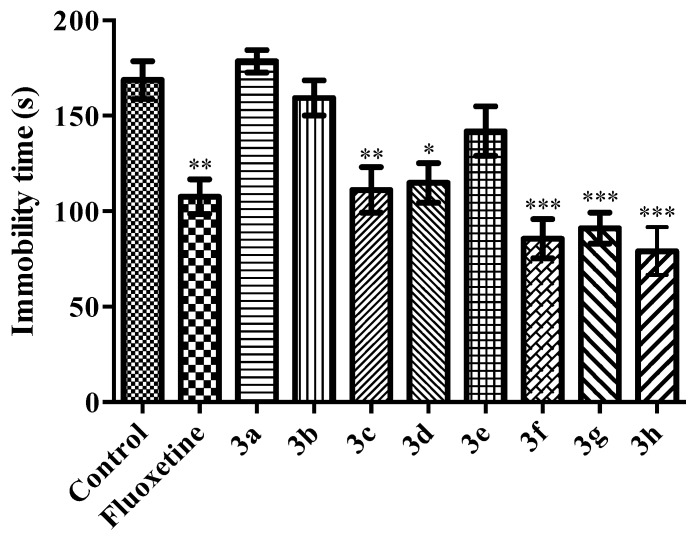
The effects of the test compounds **3a**–**3h** (40 mg/kg) and fluoxetine (20 mg/kg) on the immobility time of the mice in TST. Significant differences versus control values, * *p* < 0.05, ** *p* < 0.01, *** *p* < 0.001. Values are given as mean ± SEM, (*n* = 6).

As represented in Figure 1, the immobility time of the mice treated with compounds **3c**, **3d**, **3f**–**3h** were lower than the corresponding values of the control group in TST [F (9, 50) = 12.29; *p* < 0.001]. Fluoxetine, used as a reference drug, also reduced the immobility time of animals, as expected. However, compounds **3a**, **3b** and **3e** were ineffective in TST (Figure 1).

**Figure 2:**
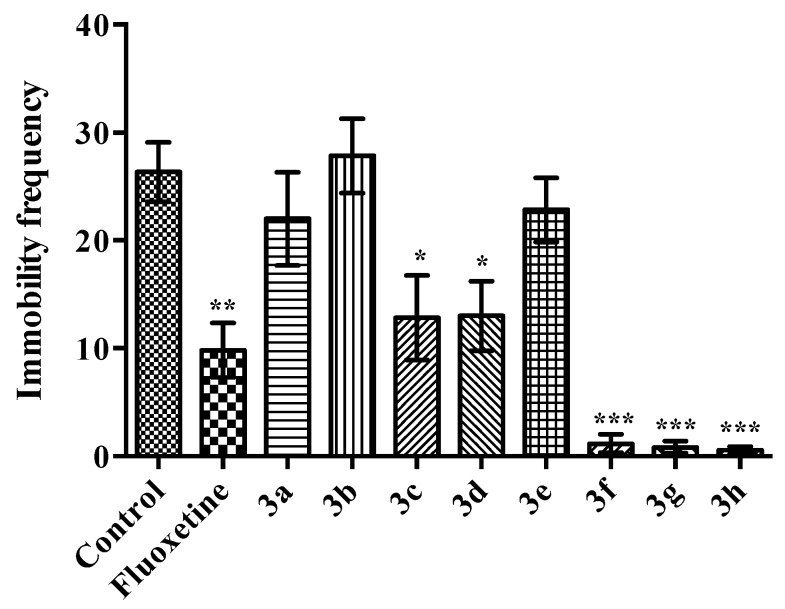
The effects of the test compounds **3a**–**3h** (40 mg/kg) and fluoxetine (20 mg/kg) on the immobility frequencies of the mice in MFST. Significant differences versus control values, * *p* < 0.05, ** *p* < 0.01, *** *p* < 0.001. Values are given as mean ± SEM, (*n* = 6).

**Figure 3:**
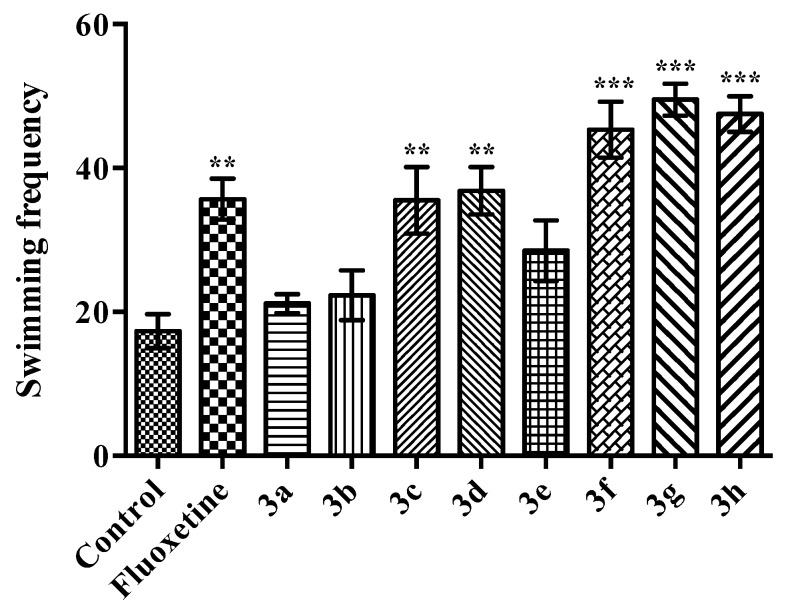
The effects of the test compounds **3a**–**3h** (40 mg/kg) and fluoxetine (20 mg/kg) on the swimming frequencies of the mice in MFST. Significant differences versus control values, ** *p* < 0.01, *** *p* < 0.001. Values are given as mean ± SEM, (*n* = 6).

**Figure 4:**
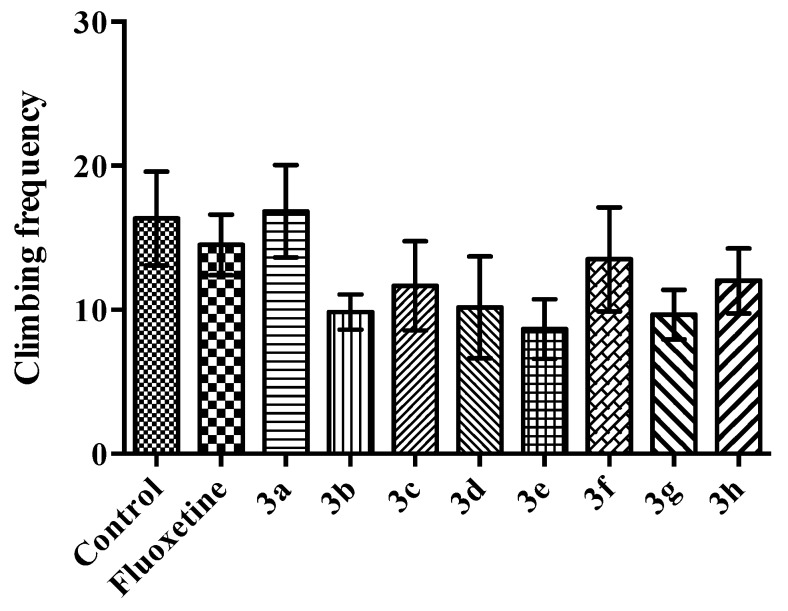
The effects of the test compounds **3a**–**3h** (40 mg/kg) and fluoxetine (20 mg/kg) on the climbing frequencies of the mice in MFST. Values are given as mean ± SEM, (*n* = 6).

As shown in Figure 2, like the reference drug fluoxetine, administration of compounds **3c**, **3d**, **3f**–**3h** significantly decreased the immobility [F (9, 50) = 14.23; *p* < 0.001] (Figure 2) but increased the swimming frequencies [F (9, 50) = 12.54; *p* < 0.001] (Figure 3) of mice without affecting the climbing behavior [F (9, 50) = 1.11; *p* > 0.05] in the MFST (Figure 4). However, compounds **3a**, **3b** and **3e** were ineffective in MFST (Figure 2, Figure 3 and Figure 4). 

**Figure 5:**
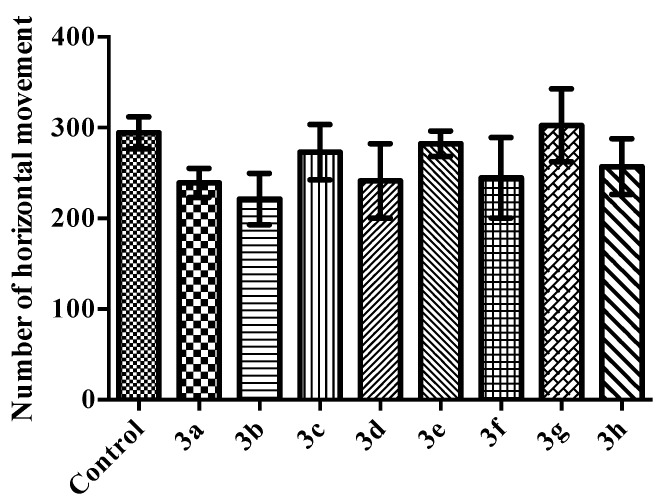
The effects of the test compounds **3a**–**3h** (40 mg/kg) on the horizontal locomotor activities of the mice in activity cage test. Values are given as mean ± SEM, (*n* = 6).

**Figure 6:**
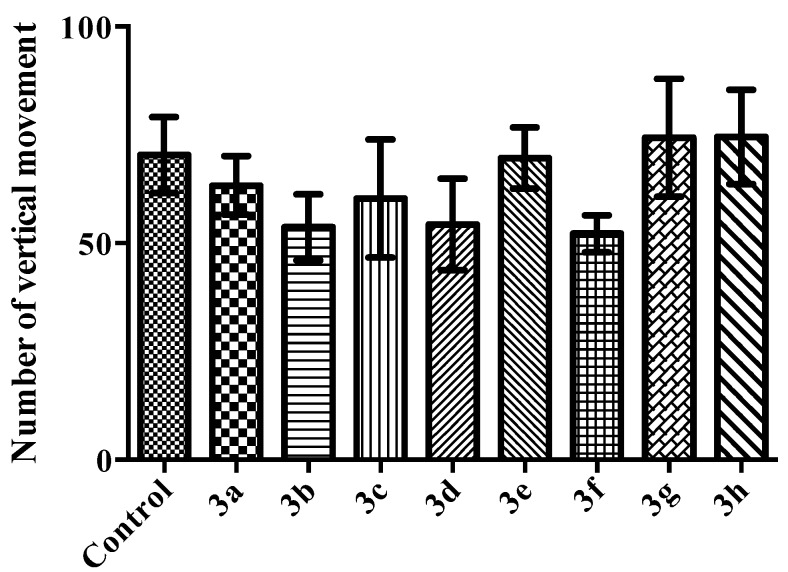
The effects of the test compounds **3a**–**3h** (40 mg/kg) on the vertical locomotor activities of the mice in activity cage test. Values are given as mean ± SEM, (*n* = 6).

Total number of horizontal [F (8, 45) = 0.79; *p* > 0.05] (Figure 5) or vertical [F (8, 45) = 0.85; *p* > 0.05] (Figure 6) spontaneous locomotor activities of the animals did not alter following the administration of the test compounds.

TST and MFST, rapid and validated animal tests, are widely used for screening the antidepressant-like activity [36]. In both of these tests, animals are exposed to a short-term and inescapable stress. Mice that are hanged from their tails in TST or forced to swim in a restricted space in MFST, initially carry out escape behaviors and then experience an “immobile posture” reflecting the behavioral despair phenomenon. The immobility behavior is accepted to associate with the depressive-like state of the animals and thus, agents reducing duration or frequency of the immobility are supposed to possess antidepressant-like effect [36,37,38,39].

In our study, in the TST and MFST, compounds **3c**, **3d**, **3f**–**3h** induced significant decreases in the duration (Figure 1) and the frequencies (Figure 2) of immobility behaviors of mice, respectively. These findings pointed out the antidepressant-like effects of compounds **3c**, **3d**, **3f**–**3h**.

MFST, superior to TST, allows to establish correlations between the “analyzed behaviors” and the “involved neurotransmitter systems”. Namely, decrease in the immobility with simultaneous increase in the swimming behavior is related to the enhanced serotonergic neurotransmission. On the other hand, agents acting on the noradrenergic system reduce immobility with a corresponding increase in the climbing behavior [36,40,41]. In this study, compounds **3c**, **3d**, **3f**–**3h**, similar to serotonergic agent fluoxetine, induces significant decrease in the immobility and increase in the swimming frequencies of mice (Figure 2 and Figure 3). These findings pointed out that serotonergic rather than noradrenergic system plays a significant role in the antidepressant-like effects of compounds **3c**, **3d**, **3f**–**3h**.

It is known that agents that alter locomotor activity counts of animals may induce false positive or false negative results in TST and MFST [42,43]. In the present study, in order to clarify whether the antidepressant-like effects of the tested compounds are associated with a possible change in the locomotor activities of mice, we performed activity cage tests. Obtained results revealed that test compounds did not induce any alteration in the total number of horizontal or vertical locomotor activities of the mice (Figure 5 and Figure 6). These data indicate that observed antidepressant-like effects of the test compounds were specific.

Most of new drug candidates fail in clinical trials owing to their toxicological profiles and reduced ADME (absorption, distribution, metabolism, and excretion) properties. The late-stage failures significantly cause to growing cost of new drug development. The capability to identify problematic issues early can intensely reduce the amount of missed time and funds, and rationalize the overall development progression. Therefore, pharmacokinetic properties of new drug candidates are extremely vital and should be assessed as early as possible in the drug development process. In this context, ADME prediction can be used to focus lead optimization to improve the preferred features of a compound [44]. 

In this study, predictions of ADME parameters of synthesized compounds **3a**–**3h** were carried out using the QikProp 4.8 software [45]. This software calculates the violations of Lipinski’s rule of five [46] and Jorgensen’s rule of three [47], which evaluate the ADME properties of new drug candidates, and is essential for the optimization of a biologically active compound. The theoretical calculations about the CNS score; number of rotatable bonds (RB) molecular weight (MW), molecular volume (MV), number of hydrogen donors (DHB), number of hydrogen acceptors (AHB), octanol/water partition coefficient (logP), aqueous solubility (logS), apparent Caco-2 cell permeability (PCaco), number of likely primer metabolic reactions (PM), percent of human oral absorption (%HOA), polar surface area (PSA) are presented in Table 1 along with the violations of rules of three (VRT) and five (VRF). According to Lipinski’s rule of five, all compounds **3a**–**3h** abide to the rules causing no violation. Moreover, these compounds meet Jorgensen’s rule of three with no more than one violation. Besides, it can be seen that all results of the rules of three and five are within the recommended ranges. CNS scores (1–2) of the compounds (Table 1) indicate that all compounds are able to cross through CNS which is essential for antidepressant drugs. However, the compounds **3a** and **3b**, carrying piperidine moiety, were inactive. This finding suggests that piperazine ring system has more contribution to antidepressant activity than piperidine moiety.

Among the piperazine bearing compounds **3c**–**3h**, compound **3e** was ineffective. Comparing the structural distance of these compounds, it was thought that extended side chain on the 4^th^ position of the piperazine may cause an activity decrease in compound **3e**. On the other hand, due to the preliminary pharmacological activity screening, a single dose (40 mg/kg) of the compounds was administered to animals and a dose-response relationship was not established. Therefore, investigating potential effect of different doses of compound **3e** may be beneficial in further studies.

Antidepressant-like potency of compounds **3c**, **3d**, **3f**–**3h** also different from each other (Figure 1 and Figure 2). Although the difference did not reach the statistical significance, compounds **3f**, **3g** and **3h**, carrying phenyl or benzyl substituents on 4th position of piperazine, seemed to be more active than compounds **3c** and **3d**. This may be caused by the better penetrance of compounds **3f**, **3g** and **3h** to the CNS; since these compounds have higher scores of logP, a predictive indicator of lipophilicity and membrane penetration, than **3c** and **3d**.

## 3. Materials and Methods

### 3.1. Chemistry

All chemicals were purchased either from Sigma-Aldrich (Sigma-Aldrich Corp., St. Louis, MO, USA) or Merck (Merck KGaA, Darmstadt, Germany) and used without further chemical purification. Melting points of the synthesized compounds were determined by a MP90 digital melting point apparatus (Mettler Toledo, Columbus, OH, USA) and were uncorrected. ^1^H- and ^13^C-NMR spectra were recorded in DMSO-*d*_6_ by a Bruker digital FT-NMR spectrometer (Bruker Bioscience, Billerica, MA, USA) at 300/75 MHz. The IR spectra were obtained on an IR Prestige-21 spectrophotometer (Shimadzu, Tokyo, Japan). LC-MS-MS studies were performed on a Shimadzu IT-TOF-LC-MS-MS system. The purities of compounds were checked by TLC on silica gel 60 F254 (Merck KGaA).

#### 3.1.1. Synthesis of 5,6-Dimethylbenzo[*d*]thiazol-2-amine (**1**)

3,4-Dimethylaniline (0.04 mol, 4.84 g) and KSCN (0.048 mol, 4.66 g) in glacial acetic acid (10 mL) were cooled in an ice bath. Bromine (0.048 mol, 2.47 mL) in glacial acetic acid (10 mL) was added dropwise at 0 °C. The mixture was allowed to stir at room temperature overnight. The iced-water (50 mL) was added in the mixture and then pH was adjusted to 11 using sodium hydroxide pellets. The precipitate was filtered, dried and recrystallized from ethanol [27].

#### 3.1.2. Synthesis of 2-Chloro-*N*-(5,6-dimethylbenzo[*d*]thiazol-2-yl) Acetamide (**2**)

5,6-Dimethylbenzo[*d*]thiazol-2-amine (**1**, 0.032 mol, 5.7 g) and triethylamine (0.038 mol, 3.85 mL) in THF (50 mL) were allowed to stir on an ice bath. Chloroacetyl chloride (0.032 mol, 3.58 g) in THF (10 mL) was added drop by drop. After this stage, the content was stirred for 1 h at room temperature. THF was evaporated and the product was recrystallized from ethanol [27,48].

#### 3.1.3. General Procedure for the Synthesis of *N*-(5,6-Dimethylbenzo[*d*]thiazol-2-yl)-2-(4-substituted piperazin/piperidine-1-yl) Acetamides **3a**–**3h**

2-Chloro-*N*-(5,6-dimethylbenzo[*d*]thiazol-2-yl) acetamide (**2**, 0.001 mol, 0.25 g), potassium carbonate (0.001 mol, 0.138 g) and appropriate piperazine/piperidine derivative (0.001 mol) were dissolved in acetone. The solution was refluxed at 40 °C for 12 h. Acetone was evaporated, residue was washed with water, filtered, dried and recrystallized from ethanol [27].

*N-(5,6-Dimethylbenzo[d]thiazol-2-yl)-2-(piperidin-1-yl) acetamide* (**3a**). Yield: 78%. m.p. 167.2 °C. FTIR (ATR, cm^−1^): 3273 (N-H), 1705 (C=O). ^1^H-NMR δ (ppm): 1.38–1.52 (7H, m, piperidine CH_2_), 2.24 (3H, s, CH_3_), 2.30 (3H, s, CH_3_), 2.45–2.51 (3H, m, piperidine CH_2_), 3.20 (2H, s, CH_2_), 7.45 (1H, s, benzothiazole, H7), 7.62 (1H, s, benzothiazole, H4), 11.84 (1H, s, NH). ^13^C-NMR δ (ppm): 19.96, 20.19, 24.10, 26.02, 54.38, 62.65, 120.89, 121.75, 129.61, 131.82, 134.54, 147.98, 159.44, 171.20 (C=O). HRMS (*m*/*z*): [M + H]^+^ calcd. for C_16_H_21_N_3_OS: 304.1466; found: 304.1478.

*N-(5,6-Dimethylbenzo[d]thiazol-2-yl)-2-(4-methylpiperidin-1-yl) acetamide* (**3b**). Yield: 83%. m.p. 252.5 °C. FTIR (ATR, cm^−1^): 3275 (N-H), 1705 (C=O). ^1^H-NMR δ (ppm): 0.88 (3H, s, CH_3_), 1.16–1.32 (3H, m, piperidine), 1.51–1.55 (2H, s, piperidine), 1.97–2.05 (2H, m, piperidine), 2.24 (3H, s, CH_3_), 2.30 (3H, s, CH_3_), 2.87 (2H, s, piperidine), 3.01 (2H, s, CH_2_), 7.20 (1H, s, benzothiazole, H7), 7.36 (1H, s, benzothiazole, H4), 11.87 (1H, s, NH).^13^C-NMR δ (ppm): 19.98, 20. 22, 21.45, 30.05, 33.17, 53.96, 62.68, 120.89, 121.75, 129.61, 131.82, 134.54, 147.98, 159.44, 171.20 (C=O). HRMS (*m*/*z*): [M + H]^+^ calcd. for C_17_H_23_N_3_OS: 318.1625; found: 318.1635.

*N-(5,6-Dimethylbenzo[d]thiazol-2-yl)-2-(4-methylpiperazin-1-yl) acetamide* (**3c**). Yield: 88%. m.p. 209.7 °C. FTIR (ATR, cm^−1^): 3277 (N-H), 1688 (C=O). ^1^H-NMR δ (ppm): 2.15 (3H, s, CH_3_), 2.21–2.33 (10H, m, piperazine CH_2_, CH_3_), 2.50 (4H, br.s., piperazine CH_2_), 3.22 (2H, s, CH_2_), 7.43 (1H, s, benzothiazole, H7), 7.60 (1H, s, benzothiazole, H4), 11.84 (1H, s, NH). ^13^C-NMR δ (ppm):19.95, 20.20, 46.26, 53.07, 55.16, 61.99, 120.80, 121.72, 129.68, 131.62, 134.41, 148.08, 158.85, 171.16 (C=O). HRMS (*m*/*z*): [M + H]^+^ calcd. for C_16_H_22_N_4_OS: 319.1576; found: 319.1587.

*N-(5,6-Dimethylbenzo[d]thiazol-2-yl)-2-(4-ethylpiperazin-1-yl) acetamide* (**3d**). Yield: 85%. m.p. 146.4 °C. FTIR (ATR, cm^−1^): 3244 (N-H), 1685 (C=O). ^1^H-NMR δ (ppm): 1.54 (3H, s, CH_3_), 2.25 (3H, s, CH_3_), 2.31 (3H, s, CH_3_), 2.32 (2H, s, CH_3_), 2.39 (4H, br.s., piperazine CH_2_), 2.50 (4H, br.s., piperazine CH_2_), 3.30 (2H, s, CH_2_), 7.53 (1H, s, benzothiazole, H7), 7.70 (1H, s, benzothiazole, H4), 11.86 (1H, s, NH). ^13^C-NMR δ (ppm):12.48, 20.01, 20.17, 52.05, 52.75, 53.12, 60.74, 121.34, 121.92, 129.28, 132.90, 135.24, 147.48, 156.96, 169.62 (C=O). HRMS (*m*/*z*): [M + H]^+^ calcd. for C_17_H_24_N_4_OS: 333.1730; found: 333.1744.

*N-(5,6-Dimethylbenzo[d]thiazol-2-yl)-2-(4-(4-fluorophenyl)piperazin-1-yl)acetamide* (**3e**). Yield: 76%. m.p. 168.5 °C. FTIR (ATR, cm^−1^): 3315 (N-H), 1701 (C=O), 831 (1,4-disubstituted benzene). ^1^H-NMR δ (ppm): 2.25 (3H, s, CH_3_), 2.32 (3H, s, CH_3_), 2.69 (4H, br.s., piperazine CH_2_), 3.12 (4H, br.s., piperazine CH_2_), 3.38 (2H, s, CH_2_), 6.92–6.97 (2H, m, fluorobenzene C-H), 7.01–7.07 (2H, m, fluorobenzene C-H), 7.53 (1H, s, benzothiazole, H7), 7.70 (1H, s, benzothiazole, H4), 12.01 (1H, s, NH). ^13^C-NMR δ (ppm): 20.01, 20.18, 49.46, 52.94, 60.61, 115.34 (d, *J* = 20.9 Hz), 117.66 (d, *J* = 7.6 Hz), 121.35, 121.92, 129.29, 131.18, 132.90, 135.25, 148.37, 156.78 (d, *J* = 234.75 Hz), 169.57, 171.21 (C=O). HRMS (*m*/*z*): [M + H]^+^ calcd. for C_21_H_23_N_4_OFS: 399.1635; found: 399.1649.

*N-(5,6-Dimethylbenzo[d]thiazol-2-yl)-2-(4-(4-fluorobenzyl)piperazin-1-yl)acetamide* (**3f**). Yield: 81%. m.p. 120.6 °C. FTIR (ATR, cm^−1^): 3238 (N-H), 1693 (C=O), 831 (1,4-disubstituted benzene). ^1^H-NMR δ (ppm): 2.24 (3H, s, CH_3_), 2.29 (3H, s, CH_3_), 2.38 (4H, br.s., piperazine CH_2_), 2.50 (4H, br.s., piperazine CH_2_), 3.23 (2H, s, CH_2_), 3.44 (2H, s, CH_2_), 7.09–7.15 (2H, m, fluorobenzene C-H), 7.30–7.34 (2H, m, fluorobenzene C-H), 7.42 (1H, s, benzothiazole, H7), 7.59 (1H, s, benzothiazole, H4), 11.84 (1H, s, NH).^13^C-NMR δ (ppm) : 19.95, 20.19, 52.97, 53.12, 61.61, 62.01, 115.30 (d, *J* = 20.9 Hz), 120.77, 121.70, 129.69, 131.05 (d, *J* = 7.9 Hz), 131.55, 134.36, 134.90 (d, *J* = 2.9 Hz), 148.10, 160.15, 161.68 (d, *J* = 240.7 Hz), 171. 27 (C=O). HRMS (*m*/*z*): [M + H]^+^ calcd. for C_22_H_25_N_4_OFS: 413.1799; found: 413.1806.

*N-(5,6-Dimethylbenzo[d]thiazol-2-yl)-2-(4-(4-chlorobenzyl)piperazin-1-yl) acetamide* (**3g**). Yield: 77%. m.p. 115.2 °C. FTIR (ATR, cm^−1^): 3238 (N-H), 1693 (C=O), 839 (1,4-disubstituted benzene). ^1^H-NMR δ (ppm): 2.25 (3H, s, CH_3_), 2.31 (3H, s, CH_3_), 2.40 (4H, br.s., piperazine CH_2_), 2.50 (4H, br.s., piperazine CH_2_), 3.30 (2H, s, CH_2_), 3.45 (2H, s, CH_2_), 7.31 (2H, d, *J* = 8.58 Hz, 1,4-disubstituted benzene), 7.37 (2H, d, *J* = 8.58 Hz, 1,4-disubstituted benzene), 7.52 (1H, s, benzothiazole, H7), 7.69 (1H, s, benzothiazole, H4), 11.85 (1H, s, NH).^13^C-NMR δ (ppm): 19.99, 20.16, 52.91, 53.02, 60.70, 61.53, 121.32, 121.90, 128.58, 129.29, 131.01, 131.86, 132.84, 135.20, 137.79, 147.50, 157.08, 169.66 (C=O). HRMS (*m*/*z*): [M + H]^+^ calcd. for C_22_H_25_N_4_OSCl: 429.1495; found: 429.1510.

*N-(5,6-Dimethylbenzo[d]thiazol-2-yl)-2-(4-(2-(dimethylamino)ethyl)piperazin-1-yl) acetamide* (**3h**). Yield: 74%. m.p. 132.1 °C. FTIR (ATR, cm^−1^): 3257 (N-H), 1693 (C=O). ^1^H-NMR δ (ppm): 2.12 (6H, s, CH_3_), 2.24 (3H, s, CH_3_), 2.31 (3H, s, CH_3_), 2.34 (2H, s, CH_2_), 2.35 (2H, s, CH_2_), 2.41 (4H, br.s., piperazine CH_2_), 2.50 (4H, br.s., piperazine CH_2_), 3.28 (2H, s, CH_2_), 7.52 (1H, s, benzothiazole, H7), 7.69 (1H, s, benzothiazole, H4), 11.85 (1H, s, NH). ^13^C-NMR δ (ppm): 19.99, 20.16, 46.01, 53.11, 53.48, 56.36, 57.08, 60.76, 121.34, 121.90, 129.29, 132.87, 135.22, 147.48, 157.01, 169.63 (C=O). HRMS (*m*/*z*): [M + H]^+^ calcd. for C_19_H_29_N_5_OS: 376.2174; found: 376.2166.

### 3.2. Prediction of ADME Parameters

In order to predict ADME parameters of synthesized compounds (**3a**–**3h)**, some physicochemical parameters were calculated by using the QikProp 4.8 software (Schrödinger, LLC, New York, NY, USA) [45].

### 3.3. Pharmacology

#### 3.3.1. Animals

Experiments were realized with adult BALB/c mice (Source: Anadolu University, Center for Animal Experiments and Research, Eskişehir, Turkey, 30–35 g). All animals were maintained under controlled environmental conditions (temperature 25 ± 1 °C, relative humidity 65% and 12 h light-dark cycle-lights on at 8:00 AM). Animals were acclimatized to laboratory conditions 24 h before the onset of experiment. The animals were maintained and treated during the experiments in accordance with the instructions of the Local Ethical Committee on Animal Experimentation of Anadolu University, Eskişehir, Turkey, which approved this study. All efforts were made to minimize animal suffering and to reduce the number of animals used in the experiments.

#### 3.3.2. Drugs and Treatments

Test compounds were administered to the mice at a dose of 40 mg/kg [33]. As test compounds were dissolved in sunflower oil, control groups received the same volume of it. In order to validate the experiments, fluoxetine (20 mg/kg, Sigma-Aldrich Chemical Company), a classical antidepressant drug, was used as a positive control. Test compounds, fluoxetine and vehicle were administrated to animals in a constant 10 mL/kg volume of body weight, through an oral gavage three times 24, 5 and 1 h before the experiments [43]. 

#### 3.3.3. Behavioral Experiments 

##### Tail Suspension Test

The TST was realized according to method described previously [39]. Acoustically and visually isolated mice were individually suspended 30 cm above the floor by their tail using adhesive tape (placed approximately 1 cm from the tip of the tail). After initial escape-oriented movements, mice hung passively and become completely motionless which is defined as immobile posture. The total duration of immobility (s) was recorded during the last 4 of 6 min test duration [49].

##### Modified Forced Swimming Test

The MFST was performed as described previously [40,50]. Mice were placed individually in a transparent glass cylinder (12 cm in diameter, 30 cm height), which was filled with water to a height of 20 cm. The temperature of the water was adjusted to 25 ± 1 °C. Two swimming sessions were performed: an initial 15-min pretest period and 24 h afterwards a 5-min test period. During the 5-min test, one of the following behavioral categories over a 5-s interval were scored [40,51]:

1. Immobility: Mouse was in an upright position on the surface with its front paws together and making only those movements required to keep the head above the water.
2. Swimming: Mouse was moving in horizontally throughout the swim chamber and crossing into another quadrant.
3. Climbing: Mouse was making vertical movements with its forepaws along the side of the swim chamber.

The water in the cylinder was changed after the test was performed for each mouse to avoid the influence of alarm substances. After both swimming sessions, the animals were removed from the cylinders, dried with towels, and returned to their home cages.

##### Activity Cage Test

The locomotor activities were evaluated individually for each animal with activity cage device (Ugo Basile, No. 7420, Varese, Italy). Parts located on two opposite sides of the device produce infrared (IR) beams. Horizontal and vertical movements of the animals were disrupted the IR light beams to the photocells and these interruptions were recorded automatically for 4 min by apparatus. The floor of the apparatus was cleaned with ethanol solution between tests [52,53].

#### 3.3.4. Statistical Analyses

All analyses were performed using the GraphPad Prism 6.01 (GraphPad Software Inc., San Diego, CA, USA). The data used in statistical analyses were acquired from 6 animals for each group. Statistical analysis of the behavioral tests was performed by analysis of variance (ANOVA), which was followed by Tukey’s post hoc comparison test. All experimental results are presented as mean (s) ± standard error of the mean (S.E.M). Probability values less than 0.05 (*p* < 0.05) were accepted as significant.

## 4. Conclusions

In this study, the antidepressant-like activities of some novel benzothiazole derivative compounds were examined using some in vivo methods. The obtained results indicated that compounds **3c**, **3d**, and **3f**–**3h** possess antidepressant-like effects in the TST and MFST, with no accompanying effect on locomotor activities of the animals. Moreover, theoretical prediction of ADME properties of the synthesized compounds indicated that all compounds seem to have good pharmacokinetic profiles. In addition to the potent intrinsic pharmacological activities of the tested benzothiazole derivatives, the good pharmacokinetic profiles of these compounds make them interesting drug candidates. However, detailed studies are required to clarify the pharmacological mechanisms underlying the observed antidepressant-like action. Moreover, comprehensive preclinical, and also clinical studies are needed to validate antidepressant efficacy of these compounds.

## Figures and Tables

**Table 1:** Some physicochemical parameters of the compounds **3a**–**3h** used in prediction of ADME profiles.

Comp	RB	CNS	MW	MV	DHB	AHB	LogP	LogS	PCaco	PM	%HOA	PSA	VRF	VRT
**3a**	3	1	303.42	1023	1	6	2.58	−3.55	729.17	5	93.28	49.84	0	0
**3b**	3	1	317.45	1056	1	6	2.78	−3.78	712.35	5	94.29	49.82	0	0
**3c**	3	2	318.44	1065	1	8	1.43	−2.01	152.72	6	74.38	57.29	0	0
**3d**	4	2	332.46	1128	1	8	1.83	−2.39	161.61	6	77.18	58.61	0	0
**3e**	3	1	398.50	1256	1	7	3.93	−5.42	680.86	5	100.00	56.25	0	0
**3f**	5	2	412.52	1309	1	8	3.33	−3.98	175.24	7	86.57	55.97	0	1
**3g**	5	2	428.98	1337	1	8	3.58	−4.35	175.24	7	88.07	55.97	0	1
**3h**	8	1	465.66	1562	1	10	3.26	−3.51	39.60	9	74.63	61.00	0	1

**RB:** Number of rotatable bonds (recommended value: 0–15); **CNS:** Predicted central nervous system activity on a –2 (inactive) to +2 (active) scale; **MW:** Molecular weight (recommended <500 g/mol); **MV:** Molecular volume in cubic angstroms using a probe with a 1.4 Å radius (recommended value: 500–2000); **DHB:** Estimated number of hydrogen bonds that would be donated by the solute to water molecules in an aqueous solution (recommended value:0–6); **AHB:** Estimated number of hydrogen bonds that would be accepted by the solute from water molecules in an aqueous solution (recommended value: 2–20); **logP:** Predicted octanol/water partition coefficient (recommended value: −2–6.5); **logS:** Predicted aqueous solubility (recommended value: −6.5–0.5 mol dm^−3^); **PCaco:** Predicted apparent Caco-2 cell permeability in nm/sec. Caco-2 cells are a model for the gut-blood barrier. QikProp predictions are for non-active transport (recommended value: <25 poor, >500 great); **PM:** Number of likely primer metabolic reactions (recommended value: 1–8); **%HOA:** Predicted human oral absorption on 0 to 100% scale. The prediction is based on a quantitative multiple linear regression model. This property usually correlates well with Human Oral-Absorption, as both measure the same property (recommended value: >80% is high, <25% is poor); **PSA:** Van der Waals surface area of polar nitrogen and oxygen atoms and carbonyl carbon atoms (recommended value: 7–200); **VRF:** Number of violations of Lipinski’s rule of five. The rules are: 7 < PSA < 200; MW < 500, logP < 5, DHB ≤ 5, AHB ≤ 10. Compounds that satisfy these rules are considered druglike. (The “five” refers to the limits, which are multiples of 5); **VRT**: Number of violations of Jorgensen’s rule of three. The three rules are: logS > −5.7, PCaco > 22 nm/s, PM < 7. Compounds with fewer (and preferably no) violations of these rules are more likely to be orally available.
